# Effect of Age and Refractive Error on the Melanopsin Mediated Post-Illumination Pupil Response (PIPR)

**DOI:** 10.1038/srep17610

**Published:** 2015-12-01

**Authors:** Prakash Adhikari, Candice A. Pearson, Alexandra M. Anderson, Andrew J. Zele, Beatrix Feigl

**Affiliations:** 1 Medical Retina and Visual Science Laboratories, Institute of Health and Biomedical Innovation, Queensland University of Technology, 60 Musk Avenue, Brisbane QLD 4059, Australia; 2 Queensland Eye Institute, South Brisbane QLD, Australia

## Abstract

Melanopsin containing intrinsically photosensitive Retinal Ganglion cells (ipRGCs) mediate the pupil light reflex (PLR) during light onset and at light offset (the post-illumination pupil response, PIPR). Recent evidence shows that the PLR and PIPR can provide non-invasive, objective markers of age-related retinal and optic nerve disease; however there is no consensus on the effects of healthy ageing or refractive error on the ipRGC mediated pupil function. Here we isolated melanopsin contributions to the pupil control pathway in 59 human participants with no ocular pathology across a range of ages and refractive errors. We show that there is no effect of age or refractive error on ipRGC inputs to the human pupil control pathway. The stability of the ipRGC mediated pupil response across the human lifespan provides a functional correlate of their robustness observed during ageing in rodent models.

The inner retinal melanopsin expressing intrinsically photosensitive Retinal Ganglion Cells (ipRGCs)^1^,^2^ control the Pupil Light Reflex (PLR)^3^,^4^ and mediate extrinsic signals from the outer retina (rods and cones)^5^. The melanopsin photopigment has a peak sensitivity at short wavelengths (~482 nm; bluish appearing light)^1^,^3^,^5^ and entirely drives the Post-Illumination Pupil Response (PIPR), the intrinsic response of ipRGCs to produce a sustained pupilloconstriction after light offset^2^,^3^,^6^,^7^. It is now known that the PIPR is affected in optic nerve and retinal diseases including glaucoma^8^,^9^,^10^, diabetes^11^, and age-related macular degeneration^12^,^13^. Given that healthy ageing can affect retinal functions including contrast sensitivity^14^, incremental light sensitivity across the visual field^15^ and dark adaptation^16^, the effect of ageing on the PIPR requires quantification before translation to clinical practice so that age-related changes can be differentiated from disease effects. However, the effect of healthy ageing on the human PIPR is not clear with two studies in eyes without disease showing opposing results. Kankipati *et al.*^17^ infer that the PIPR amplitude is independent of age using the plateau PIPR metric; Herbst *et al.*^18^ infer that the PIPR amplitude is enhanced with age using the area under curve (AUC) metric. Hence, the primary aim of this study is to determine the effect of age on the PIPR for all current metrics. To optimise the PIPR measurement for clinical use, this study also compares the PIPR amplitude and variability between the direct and consensual pupil responses, and with dilated and undilated test eyes.

Form deprivation myopia in animals is associated with a reduction in retinal dopamine^19^,^20^,^21^ and the administration of dopamine agonists inhibits this myopia development^20^,^21^,^22^,^23^ such that retinal dopamine may have antimyopiagenic effects. Outdoor light exposure and high levels of ambient illumination might be protective against myopia in children^24^,^25^,^26^, and there is speculation that ipRGC signalling may be involved in retinal dopamine production^27^,^28^ to mediate this antimyopiagenic effect. We hypothesise that if melanopsin function is associated with refractive error, and this association can be quantified with the PIPR, then the ipRGC controlled PIPR will vary with myopic refractive error. The secondary aim of this study is therefore to determine the effect of refractive error on the PIPR.

## Methods

### Participants

A total of 63 healthy participants were recruited using a purposive sampling methodology; four participants were excluded from the study because they had a cataract >Grade 2 (LOCS III) and visual acuity worse than 6/9.5. Thus, the data from 59 participants were analysed. None of the participants were taking any prescription medication known to affect the pupil light response. All 59 participants had a visual acuity of 6/9.5 or better (Bailey-Lovie Log MAR Chart), normal contrast sensitivity (Pelli-Robson Chart), normal color vision (Lanthony Desaturated D-15 Test), an intraocular pressure of <21 mmHg (tonometer, iCare Finland Oy, Helsinki, Finland), and normal central retinal thickness (RS-3000 OCT RetinaScan Advance; Nidek Co., Ltd., Tokyo, Japan). Anterior and posterior eye examination using slit lamp biomicroscopy and funduscopy revealed no pathology. Lens grading was conducted according to the Lens Opacities Classification System III (LOCS III) guideline^29^ and all participants had a lens grading of ≤2; five participants had a LOCS grade 2 cataract and eight participants had a LOCS grade 1 cataract. There was one participant with an IOL and his PIPR amplitude was in the range of the other participants. Refractive error status of the participants was obtained from an optometric examination performed within a month prior to the pupil testing. Emmetropia was considered as a mean spherical equivalent refractive error between +0.50 to −0.50 D^30^. All experiments were approved by the Queensland University of Technology (QUT) Human Research Ethics Committee (approval no: 080000546) and performed in accordance with their guidelines and regulations. The research followed the tenets of the Declaration of Helsinki and informed consent was obtained from the participants after explanation of the nature of the study.

### Pupillometer

The pupil light reflex was measured using the RAPDx pupillographer (Konan Medical USA, Inc., Irvine, CA). This instrument is designed to measure the relative afferent pupil defect (RAPD) and new customised paradigms with high irradiance stimuli were developed for measuring the PIPR. The RAPDx presents light stimuli with a 25° field of view using a liquid crystal display (LCD) with a 40 Hz frame rate. The spectral, radiometric, and photometric outputs of the stimuli were measured with a Spectroradiometer (StellarNet, Florida, USA) and an ILT1700 Research Radiometer (International Light Technologies, Massachusetts, USA). The monochromatic stimuli included 1 s and 10 s pulses of blue light (short wavelength, λ_max_ = 448 nm, corneal irradiance: 14.5 log quanta.cm^−2^.s^−1^, luminance: 1.3 log cd.m^−2^) with high melanopsin excitation (609.8 α-opic lux) and red light (long wavelength, λ_max_ = 604 nm, corneal irradiance: 14.4 log quanta.cm^−2^.s^−1^, luminance: 1.0 log cd.m^−2^) with low melanopsin excitation (9.7 α-opic lux). Blue light measures the intrinsic melanopsin response and red light measures the outer retina response and serves as a control (see Quantification of the PLR and PIPR section for details). During pupil recording, the participants fixated on a green cross in the center of the field at infinity through a pair of 50 mm objective lenses (Konan Medical USA, Inc., Irvine, CA). The test stimulus was presented in Newtonian view to one eye and the PLR of both eyes was recorded under infrared illumination.

### Pupillometry protocol

**Figure 1:**
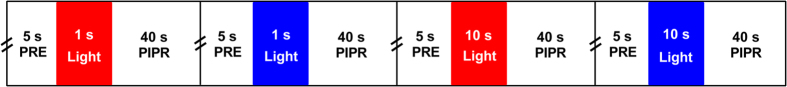
Stimulus protocol for pupillometry. Red stimuli (red rectangles) and blue stimuli (blue rectangles) were alternated. The double slash before each 5 s pre-stimulus duration indicates a 10 s interval. Measurements were repeated twice; a two minutes break was given between the repeats of this sequence. PRE = pre-stimulus duration; PIPR = post-illumination pupil response.

All measurements were preceded by 10 minutes dark adaptation in our laboratory (<6 lux). Testing with 1 s light pulses always preceded the 10 s pulses (Fig. 1). Red and blue stimuli were alternated in all sessions to control for the effect of melanopsin bistability^31^. The order of stimulus presentation was therefore: 1 s red, 1 s blue, 10 s red, and 10 s blue. With every stimulus, the baseline pupil diameter was measured in the dark during 5 s of fixation before the onset of light pulse (1 s or 10 s) and the PIPR was recorded for 40 s after stimulus offset. The PLR was measured under two conditions: 1) Undilated; one eye was stimulated and both the direct and consensual PLR were recorded and 2) dilated; the stimulated eye was dilated with 1% Tropicamide (Minims, Chauvin Pharmaceuticals Ltd., England) and the consensual PLR in the fellow eye was recorded. The dilated pupil was randomly selected. Measurements were repeated two times. Participants were tested between 10 AM and 5 PM to minimise the effect of circadian variation on the PIPR amplitude^32^,^33^.

### Quantification of the PLR and PIPR

**Figure 2:**
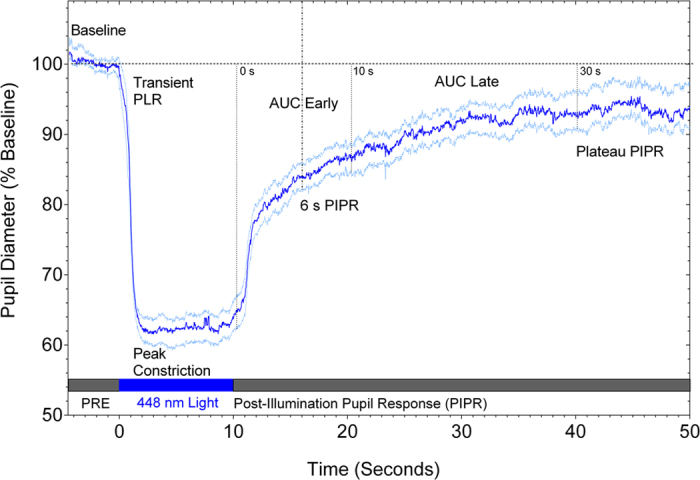
Pupil trace showing the average (n = 59 participants; 21–70 years old) pupil light reflex (PLR) during presentation of a 10 s, 448 nm (blue) light pulse (14.5 log quanta.cm^−2^.s^−1^; 25° diameter), and the post-illumination pupil response (PIPR) after light offset. The dark blue trace shows the mean PLR and PIPR and light blue traces show the 95% confidence limits of the mean. The temporal sequence of the pupillometry protocol is indicated by the filled rectangles positioned along the abscissa. The pupil analysis metrics are noted on the trace and defined in Table 1. PRE = pre-stimulus duration; AUC = area under curve.

The PLR during light stimulation was quantified using the transient PLR and peak pupil constriction metrics described in Table 1 and Fig. 2. The transient PLR is predominantly controlled by the outer retina, at least at long wavelengths^34^ and the peak pupil constriction has been shown to quantify the combination of outer and inner retinal inputs depending on stimulus wavelength^4^. To determine inner retinal ipRGC function, the PIPR after light offset was quantified with the four metrics (6 s, plateau, AUC early and late) (see Feigl *et al.*^12^ and Adhikari *et al.*^7^ for details of the metrics). The metrics were derived from the best-fit of the linear and exponential models to the data^6^,^8^,^11^,^32^. The baseline pupil diameter (BPD) decreases with age^17^,^35^,^36^ and it affects the pupil constriction amplitude (in mm) such that a smaller amplitude is observed with a smaller BPD^17^. To account for this effect, the pupil diameter during light stimulation and after light offset was normalised to the BPD. The peak pupil constriction and PIPR amplitudes are therefore presented in percentage of the BPD. For the peak constriction amplitude, 6 s PIPR, and plateau PIPR, a smaller percentage value indicates a larger pupil response.

### Statistical analysis

Statistical data analysis was conducted using GraphPad Prism (GraphPad Software, Inc., CA, USA). The effect of age on the PLR and PIPR metrics was the primary outcome and the effect of refractive error on the metrics was the secondary outcome. Age and refractive error were independent (predictor) variables and the PLR and PIPR metrics given in Table 1 and Fig. 2 were dependent (outcome) variables. The confounding variables due to age-related changes in baseline pupil diameter (BPD) and lenticular opacity were controlled by normalising the pupil data to the BPD, including a measurement of the consensual PLR with light presented to the dilated fellow eye and excluding the participants with cataract >Grade 2, respectively (see Participants and Quantification of the PLR and PIPR sections). Shapiro-Wilk test showed all PLR and PIPR metrics were normally distributed and thus statistical analyses of the effect of age on pupil metrics were performed with simple linear regression models. The statistical significance of this effect was determined on the basis of whether or not the slope of the best-fitting linear regression line was significantly different from zero using *F*-test (95% confidence interval, *P* < 0.05). Paired *t*-test was used to calculate the difference in the PLR and PIPR amplitudes between outcome variables (10 s vs 1 s pulses, direct vs consensual PLR, dilated vs undilated) and the difference in the 6 s PIPR amplitude between blue and red stimuli (*P* < 0.05). To quantify the variability of the PIPR metrics, the intra- and inter-individual Coefficient of Variation (CV) was calculated as Standard Deviation (SD) divided by the Mean. Intra-individual CV was based on the difference between the repeats for the same participant and inter-individual CV was based on the difference among all participants. The difference in intra-individual CV among the PIPR metrics was tested with one-way ANOVA (95% confidence interval, *P* < 0.05, Turkey’s test for post-hoc analysis, Geisser-Greenhouse correction). To determine the effect of refractive status on the PLR and PIPR amplitudes, the difference in the PLR and PIPR amplitudes among participant sub-groups classified according to refractive status was tested with Kruskal-Wallis test (Geisser-Greenhouse correction) because the refractive error data distribution was non-normal. Due to low sample number with myopia (n = 13) and hyperopia (n = 3), the refractive error and pupil data were bootstrapped using Microsoft Excel 2010 (Microsoft, Redmond, WA, USA)^37^. Thirty nine original samples were used to create 1560 bootstrap replication estimates according to the recommendation by Davison and Hinkley^38^. Refractive error bootstrapped estimates ranged from −2.25 D to +0.25 D, representing 84.6% of the original sample.

## Results

**Figure 3:**
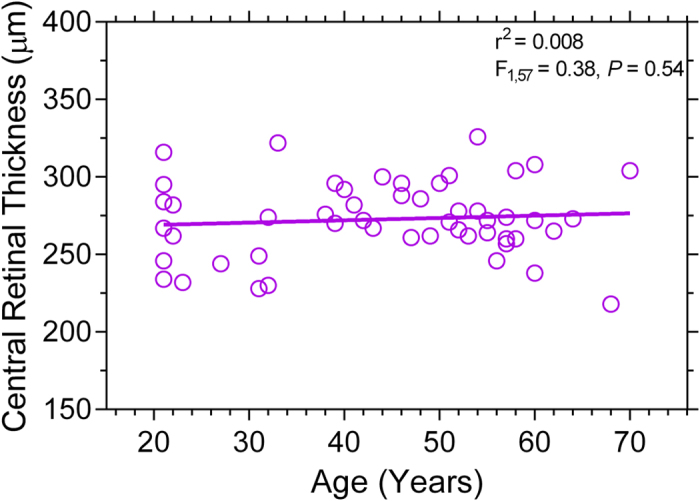
Relationship between age and the central retinal thickness (n = 59 participants). The solid line indicates the best-fitting linear regression. The *F*-value indicates the slope of the regression line does not change as a function of age.

A total of 59 participants (40 female, 19 male) were included in this study with an age range between 21 to 70 years (mean age ±SD: 43.7 ± 14.4 years) and there was equal distribution of participants in each age decade. The mean ± SD central retinal thickness was 272.72 ± 24.02 μm and the slope of the regression line (Fig. 3) was not significantly different from zero indicating that the central retinal thickness is independent of age in agreement with literature reports^39^,^40^.

Table 2 presents the descriptive data on the effect of outcome variables on the PLR and PIPR; the 10 s pulses produce both larger peak pupil constriction and PIPR amplitudes than 1 s pulses with blue lights under fellow eye pupil dilation when the stimuli are presented in Newtonian view. The undilated direct and consensual pupil responses for the PLR and PIPR metrics with 10 s pulses were not significantly different. With fellow eye pupil dilation, the consensual PLR and PIPR amplitudes were significantly larger than with undilated pupils as expected. The intra- and inter-individual Coefficients of Variation (CV) however, were independent of the testing conditions (10 s and 1 s light pulses, direct and consensual response, and dilated and undilated conditions). Due to commonality of the patterns of the 1 s and 10 s data as well as the dilated and undilated pupil data, the following analyses focus on the consensual pupil response to the 10 s stimulus presented to the fellow dilated eye.

**Figure 4:**
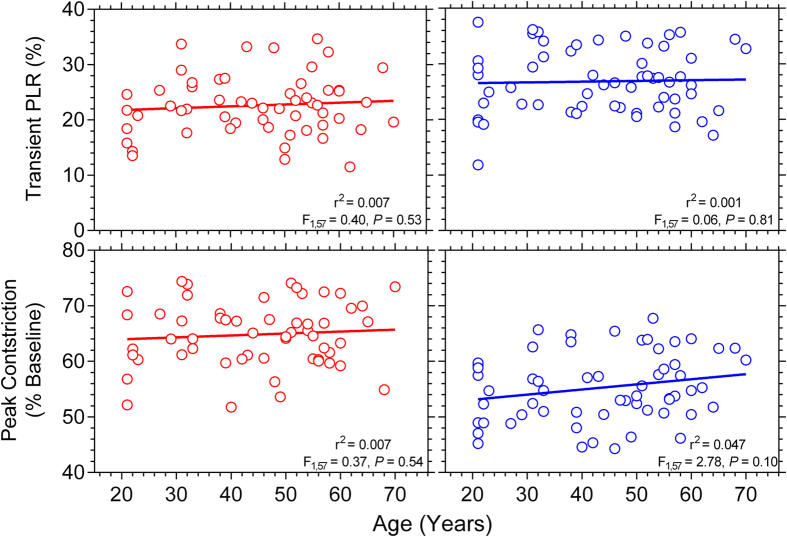
Relationship between age and the transient PLR (upper panels) and peak pupil constriction (lower panels) (n = 59 participants). The red and blue circles indicate the response with red and blue lights, respectively; and the solid lines show the best-fitting linear regressions. The *F*-values indicate the slopes of the regression lines do not change as a function of age.

**Figure 5:**
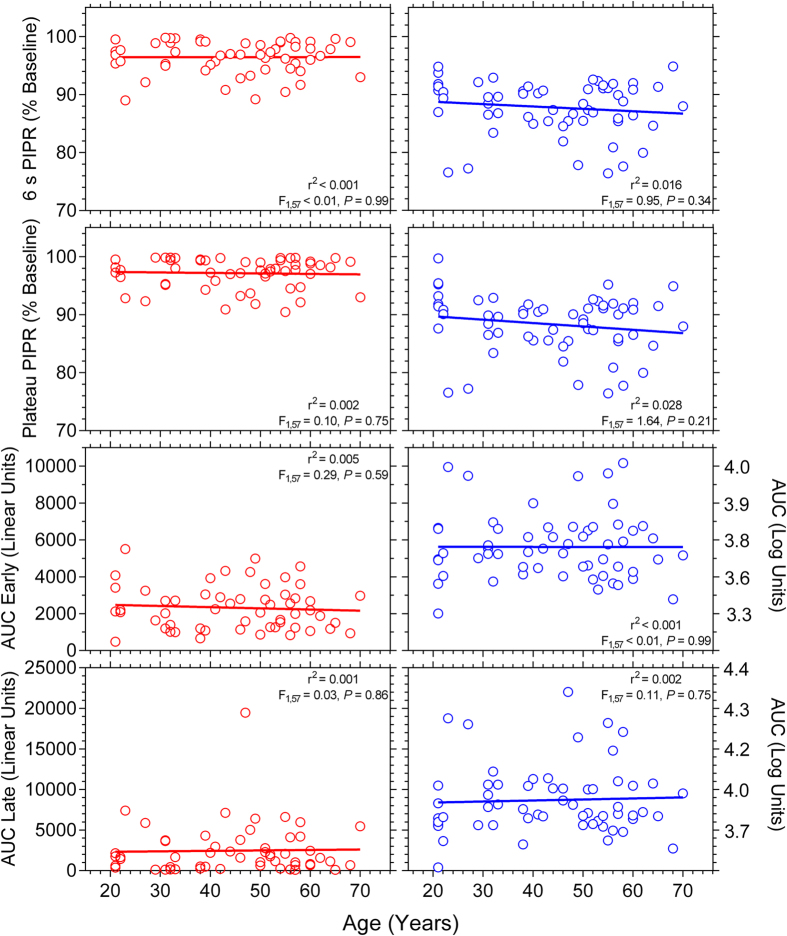
Relationship between age and the PIPR metrics (6 s PIPR, Plateau PIPR, AUC Early, and AUC Late recovery) (n = 59 participants). The red and blue circles indicate the response with red and blue lights, respectively; and the solid lines show the best-fitting linear regressions. The *F*-values indicate the slopes of the regression lines do not change as a function of age.

During light stimulation, the slopes of the regression lines describing the transient PLR and peak pupil constriction data were not significantly different from zero for red and blue lights indicating that these metrics are independent of age (Fig. 4). We determined the relationship between age and the Post-Illumination Pupil Response (PIPR) for four PIPR metrics (Fig. 5). Blue pulses produced a significantly larger 6 s PIPR amplitude than red pulses (Paired *t*-test: *t*(57) = 20.07, *P* < 0.001) due to the higher melanopsin excitation with the short wavelength (blue) light. The slopes of the regression lines were not significantly different from zero for any PIPR metric indicating the PIPR amplitude is independent of age.

**Figure 6:**
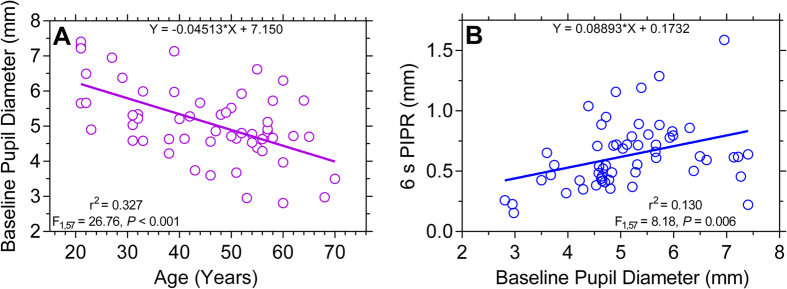
Relationship between the baseline pupil diameter (mm) and age (Panel (A)) and the 6 s PIPR (Panel (B)) (n = 59 participants). The 6 s PIPR is given in mm (not % baseline as in the other figures) and a larger value indicates a larger PIPR. The *F*-values indicate the slopes of the regression lines are significantly different from zero.

The baseline pupil diameter (BPD) measured in the dark decreases significantly with age in agreement with previous studies (Fig. 6A)^17^,^35^,^36^. The BPD decreases during ageing by 0.045 mm per year, similar to a previously reported reduction rate of 0.043 mm per year^36^. We further confirm a previous report^17^ that the PIPR amplitude with blue lights increases significantly with increasing BPD (r^2^ = 0.130, *P* = 0.01), validating the use of percentage (%) BPD to describe the PLR and PIPR metrics (Fig. 6B).

**Figure 7:**
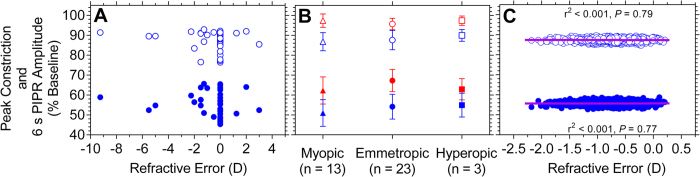
Panel (A) Scatterplot showing the peak pupil constriction (filled blue circles) and 6 s PIPR amplitude (open blue circles) for blue light as a function of refractive error (spherical equivalent, Dioptre, D). Panel (**B**): Median ± SD peak pupil constriction (filled symbols) and 6 s PIPR amplitude (open symbols) with red (red symbols) and blue (blue symbols) light pulses for myopes (triangles), emmetropes (circles), and hyperopes (squares). Panel (**C**): Scatterplot showing the bootstrapped estimates (B = 1560) of the data in Panel A; the solid lines indicate the best-fitting linear regressions.

The refractive status of 39 participants was known and the refractive error ranged from +3.00 D to −9.25 D (spherical equivalent). Due to a small sample of refractive errors, we bootstrapped the data to obtain an estimate of the sampling distribution for regression analysis (see Statistical analysis). The slopes of the best-fitting linear regression lines to the bootstrapped data (Fig. 7C) were not significantly different from zero indicating that the peak constriction and 6 s PIPR metrics have no relationship to refractive error. To determine the effect of refractive error status on the PLR and PIPR amplitudes, the participants were divided into three sub-groups on the basis of refractive error: emmetropes (< ±0.5 D) (n = 23), hyperopes (≥ +0.5 D) (n = 3), and myopes (≥−0.5 D) (n = 13) (Fig. 7B). Between the sub-groups, there was no significant difference in the peak pupil constriction amplitude (Kruskal-Wallis test; blue light: H = 0.77, *P* = 0.68; red light: H = 0.35, *P* = 0.84) or the 6 s PIPR amplitude (blue light: H = 1.84, *P* = 0.40, red light: H = 4.36, *P* = 0.11). The transient PLR (data not shown) was also unaffected by refractive status.

**Figure 8:**
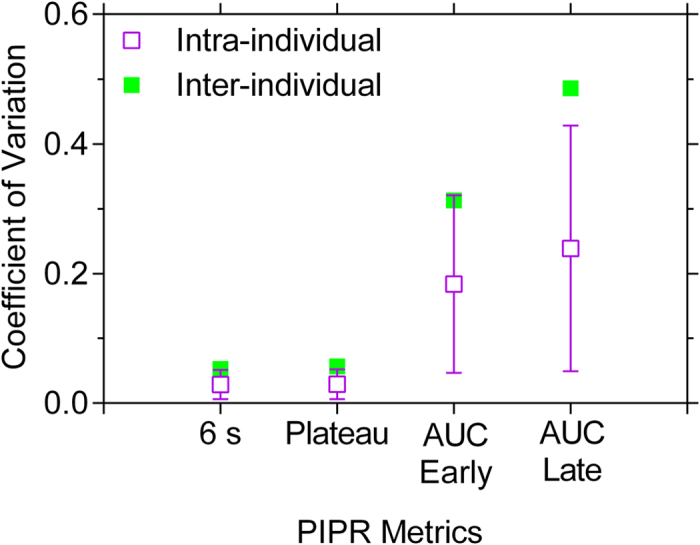
Mean ± SD (n = 59 participants) intra- and inter-individual Coefficient of Variation (CV) of the PIPR metrics with blue light; red light showed similar results (not shown).

To consider the variability of the PIPR metrics, the intra- and inter-individual Coefficient of Variation (CV) was calculated (Fig. 8). The intra-individual CV significantly differed among the four PIPR metrics (One-Way ANOVA: *F*_3,196_ = 41.45, *P* < 0.001). Post-hoc analysis indicated that the intra-individual CV for the 6 s and plateau metrics was significantly different to the AUC metrics, with a lower variation for the 6 s and plateau metrics compared to the AUC metrics (Fig. 8). Similarly, inter-individual CV was lower for the 6 s and plateau metrics compared to the AUC metrics.

## Discussion

The primary outcome of this study shows that ageing has no effect on the Post-Illumination Pupil Response (PIPR) indicating that the intrinsically photosensitive Retinal Ganglion Cell (ipRGC) inputs to the pupil control pathway show no change with age. The 6 s and plateau PIPR metrics demonstrated the lowest intra- and inter-individual coefficient of variation. The secondary outcome indicates that the PIPR is independent of refractive status.

This is the initial study showing the PIPR amplitude (quantified with all current PIPR metrics: 6 s PIPR, plateau PIPR, AUC early and late recovery), which is entirely driven by the intrinsic response of melanopsin expressing ipRGCs, is independent of age (Fig. 5). Kankipati *et al.*^17^ showed that the PIPR quantified with the plateau metric is independent of age. On the other hand, Herbst *et al.*^18^ attributed the enhancement of the PIPR with ageing measured via the AUC metrics to increased light scattering in the ageing lens such that this scattering might excite more ipRGC axonal collaterals thereby leading to a larger PIPR amplitude. In this study, the exclusion criteria limited the confounding effect of lenticular scattering on ipRGC stimulation by excluding participants with lens opacity >Grade 2 on LOCS III. Although the AUC early and late metrics have higher variability compared to 6 s and plateau metrics^7^, we observed in our study with a larger sample than the previous two that the PIPR amplitude was independent of age for all PIPR metrics. Our finding of no age effect on ipRGC input to the pupil is supported by a recent study in healthy rats showing the robustness of ipRGC density and dendritic arborisation to ageing^41^. In addition, ipRGCs input to the suprachiasmatic nucleus (SCN) for circadian photoentrainment and it is known that the number of the SCN neurons in the rhesus monkey is preserved during healthy ageing^42^. As such we infer that the neuronal function of the olivary pretectal nucleus (OPN), which is the relay to the PLR pathway, may be also preserved with ageing, thus stabilising the PLR and PIPR amplitudes throughout adulthood.

In accordance with Kankipati *et al.*^17^, we observed that the PIPR increases with increasing baseline pupil diameter (BPD), which decreases with age and thus affects the PIPR metrics. We therefore reiterate the importance of normalising pupil data to BPD to minimise the variability in the PLR and PIPR incurred due to variable BPD. With the Newtonian view used in the commercial pupillometer, the 6 s and plateau metrics are more robust to variation than other PIPR metrics in agreement with our recent study using a custom built Maxwellian view pupillometer^7^.

It has been suggested that reduction in light stimulated pupil size (in mm) with age is related to decrease in BPD due to iris atrophy and impaired sympathetic nerve supply to iris with age^43^. However, when normalised to BPD, there should be no effect of healthy ageing on the peak pupil constriction amplitude as our data demonstrate (Fig. 4). The transient PLR receives predominant inputs from the outer retina, at least at long wavelengths^34^ where melanopsin has low sensitivity^3^,^6^,^7^, and this transient response showed no significant change with age for either wavelength. This finding indicates that outer retina inputs to the PLR, which are mediated extrinsically to the OPN via ipRGCs, are not affected by age either. Histological studies in humans demonstrate that while central retinal cone density remains stable during adulthood and central retinal rod density deteriorates with ageing^44^, peripheral retinal rod cell density also remains stable throughout adulthood^44^. As our stimulus was 25° diameter, any deficit in central retinal rods (if present) in older persons would have been masked by the contributions from healthy peripheral rods (and cones) because the PLR has large spatial summation^45^. A smaller stimulus may be more sensitive to demonstrate such focal defects^12^.

Earlier studies investigating the relationship of refractive error and the light-adapted pupil size demonstrate either a weak negative correlation (pupil size is larger in myopes and smaller in hyperopes than in emmetropes)^46^ or no correlation^36^. However, these studies did not determine the PLR or PIPR and refractive error. Light exposure has been associated with myopia development^25^,^26^ and thus ipRGCs have been speculated to be responsible for the production of retinal dopamine^27^,^28^ that may have antimyopiagenic effects^19^,^20^,^22^; but no study has evaluated the relationship between myopia and ipRGC function in humans. We present the first report that refractive error (+3.00 D to −9.25 D) has no effect on the PLR during light stimulation (described by peak pupil constriction) as well as on the PIPR after light offset (described by 6 s PIPR amplitude) (Fig. 7). We could not control for pre-light exposure because we had a cohort with a wide age range, and the measurement of a short period of pre-exposure to light (e.g. during a two week period monitored with actigraphy) is not likely to be representative of long term light exposure, nor is there evidence of short duration light exposures causing permanent myopic changes in humans.

We found no significant difference between direct and consensual PLR and PIPR amplitudes using monochromatic 1 s and 10 s blue and red light pulses in the central 25° (Table 2), although a 6.8% difference between direct and consensual PLR amplitudes has been reported in normal population (n = 59 participants) using 500 ms white light pulses (1.8 mm diameter in the pupil plane) stimulating the nasal retina^47^. Here, we did not observe a significant difference in variability of direct and consensual responses, indicating the consensual PLR and PIPR measurement is equally valid as the direct PLR and PIPR measurement, at least in our sample. With the current Newtonian view pupillometer, the PLR and PIPR amplitudes measured under dilation are larger than with undilated pupil as would be expected due to the higher retinal irradiance^48^. Thus, we recommend that with a Newtonian view pupillometer, the stimulated pupil should be dilated to maximise the retinal irradiance and PIPR amplitudes for effective differentiation between healthy and diseased eyes in a clinical setting. We demonstrate that 10 s pulses produce slightly (1.7%) larger PIPR amplitudes than 1 s pulses. This difference is contextually non-significant considering 0.03 to 0.04 (3% to 4%) variation in the PIPR indicating the PIPR amplitude does not increase with increasing stimulation duration from 1 s to 10 s due to melanopsin adaptation over time, in agreement with our study^7^. Moreover, this small difference can be attributed to a lower corneal irradiance (14.5 vs 15.1 log quanta.cm^−2^.s^−1^) and shorter dominant wavelength (448 nm) than melanopsin peak sensitivity in the commercial pupillometer compared to our recent study^7^ with a custom Maxwellian view optical system (dominant wavelength = 465 nm).

In conclusion, this is the first study showing that ipRGC function measured with all current PIPR metrics is unaffected by ageing thus providing a functional correlate of their robustness throughout lifespan as observed in rodent *in vivo* models. We further present the initial and novel findings suggesting that the ipRGC controlled PIPR is also independent of refractive error.

## Additional Information

**How to cite this article**: Adhikari, P. *et al.* Effect of Age and Refractive Error on the Melanopsin Mediated Post-Illumination Pupil Response (PIPR). *Sci. Rep.* **5**, 17610; doi: 10.1038/srep17610 (2015).

## Figures and Tables

**Table 1:** Definitions for the PLR metrics during light stimulation and PIPR metrics after light offset (following Adhikari *et al.*, 2015^7^).

Metrics	Definition and Units
Baseline pupil diameter (BPD)	Average 5 s pre-stimulus period (mm, %)
**PLR Metrics**	
Transient PLR	Peak % change from 180–500 ms after light onset^11^,^34^
Peak pupil constriction	Minimum pupil size (% baseline) during light presentation
**PIPR Metrics**	
6 s PIPR amplitude	Pupil size (% baseline) at 6 s after light offset^8^,^32^,^49^
Plateau PIPR	Plateau of exponential model (% baseline)^8^
AUC early	∑ (BPD - APD)* over 0–10 s after light offset (unitless)^50^
AUC late	∑ (BPD - APD) over 10–30 s after light offset (unitless)^50^

*APD = absolute pupil diameter.

**Table 2:** Comparison of the PLR and PIPR amplitudes and Coefficients of Variation (CV) with blue lights between outcome variables: 10 s and 1 s pulses, direct and consensual pupil response, and dilated and undilated pupils.

	*Peak Pupil Constriction*			*6 s PIPR*		
	Amplitude (% Baseline)	Intra-individual CV	Inter-individual CV	Amplitude (% Baseline)	Intra-individual CV	Inter-individual CV
Consensual 10 s vs 1 s (Dilated)	55.28 ± 6.15 vs 61.11 ± 5.32 (*P* < 0.001)*	0.04 ± 0.03 vs 0.05 ± 0.06 (*P* = 0.47)	0.09 vs 0.10	92.74 ± 3.36 vs 91.55 ± 3.64 (*P* < 0.001)*	0.03 ± 0.03 vs 0.03 ± 0.02 (*P* = 0.44)	0.04 vs 0.04
Direct vs Consensual 10 s (Undilated)	57.22 ± 7.22 vs 57.97 ± 6.00 (*P* = 0.70)	0.07 ± 0.20 vs 0.05 ± 0.06 (*P* = 0.31)	0.12 vs 0.10	91.65 ± 3.59 vs 91.55 ± 3.64 (*P* = 0.68)	0.03 ± 0.03 vs 0.03 ± 0.02 (*P* = 0.06)	0.04 vs 0.04
Dilated vs Undilated 10 s (Consensual)	55.28 ± 6.15 vs 57.97 ± 6.00 (*P* < 0.001)*	0.05 ± 0.06 vs 0.05 ± 0.06 (*P* = 0.94)	0.11 vs 0.10	87.78 ± 4.62 vs 91.55 ± 3.64 (*P* < 0.001)*	0.03 ± 0.02 vs 0.03 ± 0.02 (*P* = 0.36)	0.05 vs 0.04

*Statistically significant.
